# Tutorial: unified 1D inversion of the acoustic reflection response

**DOI:** 10.1111/1365-2478.12946

**Published:** 2020-03-17

**Authors:** Evert Slob, Kees Wapenaar, Sven Treitel

**Affiliations:** ^1^ Department of Geoscience and Engineering Delft University of Technology P.O. Box 5048 Delft GA 2600 The Netherlands; ^2^ Tridekon Oklahoma USA

**Keywords:** acoustic, inversion, numerical study

## Abstract

Acoustic inversion in one‐dimension gives impedance as a function of travel time. Inverting the reflection response is a linear problem. Recursive methods, from top to bottom or vice versa, are known and use a fundamental wave field that is computed from the reflection response. An integral over the solution to the Marchenko equation, on the other hand, retrieves the impedance at any vertical travel time instant. It is a non‐recursive method, but requires the zero‐frequency value of the reflection response. These methods use the same fundamental wave field in different ways. Combining the two methods leads to a non‐recursive scheme that works with finite‐frequency bandwidth. This can be used for target‐oriented inversion. When a reflection response is available along a line over a horizontally layered medium, the thickness and wave velocity of any layer can be obtained together with the velocity of an adjacent layer and the density ratio of the two layers. Statistical analysis over 1000 noise realizations shows that the forward recursive method and the Marchenko‐type method perform well on computed noisy data.

## INTRODUCTION

Backus (1959) showed that the reflections in a marine seismic trace can be understood as the subsurface reflection response filtered by a water layer reverberation operator that is independent of the source and receiver depth. He also showed how a simple three‐term deconvolution filter can be constructed and used to remove these water‐layer reverberations. Kunetz (1964) showed that both free‐surface and internal multiples from the shallow part of the reflection response can be filtered from the data. Application of this filter removes overlap from multiples and the first deeper primary reflection which can then be analysed. He showed that the filter consists of two parts of a fundamental wave field and gave their mutual recurrence relation. Combining the filter and the recurrence relations leads to a forward recursive inversion scheme. It successively filters from top to bottom the local reflection coefficients from the data. The same idea, but using an energy equation, was used by Robinson (1967) and Robinson and Treitel (1977) to construct a similar forward recursive scheme. They also showed that the filters at each step can be used to compute the up‐ and down‐going wave fields at depth from the surface reflection response. Robinson and Treitel (1978) showed how the energy relation can be used to construct a scheme that starts in the bottom of a model. It leads to a backward recursive scheme to obtain the local reflection coefficients. All the work was done with the Goupillaud's (1961) model of a discrete‐layered medium. In these three schemes, one equation is used that involves the data. Kunetz (1964) used the convolutional model, whereas Robinson and Treitel (1977, 1978) used an energy relation. The other equations are the recursive relations of the two parts of the fundamental wave field to make a forward or backward recursion step. The reflection coefficient is retrieved as the amplitude of an event at the proper two‐way travel time, which is then also found. From the refection coefficients, the layer impedances are found and this completes the inversion.

Many people worked on the exact inverse solution of the Schrödinger equation to resolve the scattering potential from the scattered field (Agranovich and Marchenko 1963; Lamb 1980). This solution was taken by Ware and Aki (1969) who showed that if depth was converted to vertical travel time and the fields were flux‐normalized, the one‐dimensional acoustic wave equation is equal to the Schrödinger equation. The inverse solution consists of a time integral from which the fundamental reflection solution can be computed. The acoustic impedance at any fixed vertical travel time is obtained by integrating the fundamental reflection solution over all times. It requires the zero‐frequency component to be recorded in the seismic record (Berryman and Greene 1980). Rose (2002) showed how we can understand the solution to the Marchenko equation as creating a focused wave field at depth. This led Broggini, Snieder and Wapenaar (2012) to the understanding that this focus leads to a response at the acquisition surface as coming from a virtual source at the focal point. By reciprocity, this is the same as the understanding of finding the wave field at depth from a source at the acquisition surface by Robinson and Treitel (1977). Slob *et al*. (2014) showed that the fundamental reflection solution of the Marchenko equation is a particular combination of the up‐ and down‐going parts of the fundamental wave field. They showed how the Marchenko equation can be seen as a sum of two other equations. One that represents a seismic experiment and one that represents a time‐reversed seismic experiment. They derived these two equations by applying acoustic reciprocity of the time‐convolution and time‐correlation types. They showed that the local reflection coefficient at any vertical travel time instant is obtained directly from the up‐going part of the fundamental wave field. Wapenaar *et al*. (2013) derived the single‐sided Marchenko equation for a three‐dimensional heterogeneous medium, and connected the concepts of Marchenko inverse scattering and Green's function retrieval. This triggered new research on redatuming, imaging and inversion with minimal model information and multiple elimination without model information. Review and tutorial papers have been published that help getting into the subject of Marchenko redatuming and imaging (Wapenaar *et al*. 2017; Nowack and Kiraz 2018; Lomas and Curtis 2019).

Bardan and Robinson (2018) connect the result of the forward recursive scheme of Kunetz (1964) to the solution of the discrete version of the Marchenko equation from Berryman and Greene (1980). They conclude that the discrete solution of Kunetz (1964) is the same as that of the discrete Marchenko equation. Here, we show that the two bodies of thought described above are connected through the fundamental wave field. The fundamental wave field as derived in discrete forms of the recursive schemes has up‐ and down‐going parts and together make up the fundamental reflection solution of the Marchenko equation. We show how the equations that are used for the forward and backward recursive schemes can be combined at any depth level to yield the two parts of the Marchenko equation. We then show how the combination of the two equations removes the need for recursive solutions. Only in this way the inversion can be done at any chosen vertical travel time in a target‐oriented way for finite‐frequency‐bandwidth data. We show how the reflection response along a line over a horizontally layered medium leads to the possibility of obtaining layer thickness and velocity of a target layer, together with the velocity of the two adjacent layers and the density ratios of these layers.

First, we give the expressions for a two‐sided experiment to build the necessary expressions for the impulse reflection and transmission responses of a layered medium. We establish the convolutional model and the time‐reversed experiment that lead to the energy relations. The subsurface impulse reflection response is represented by the fundamental wave field and we give the recurrence relations between the up‐ and down‐going parts. We derive Green's function representations in terms of the reflection response and the fundamental wave field. From these representations, we derive the Marchenko equation. Second, we derive four inversion methods and show that they are based on the same set of equations. We show how the inversions are carried out and which information is obtained. Third, we show how only the inversion with the Marchenko‐type method can be extended to target‐oriented inversion of finite‐frequency‐bandwidth data. Finally, we give a numerical example to illustrate the performance of the methods on noisy data and evaluate the results.

## THE CONVOLUTIONAL MODEL OF ACOUSTIC EXPERIMENTS

In this section, we describe the discrete‐layered model and the associated wave fields that can be measured on both sides of the model given a source in the top or in the bottom. Without loss of generality, we model the acquisition surface as a transparent boundary in the upper half space and in the lower half space. Inclusion of a free surface would not add an unknown reflector as explained by Kunetz (1964). We use the physics of acoustic wave propagation in a linear model, which says that the response of a medium is the convolution of the time signature of the source and the earth impulse response. Later, we use this understanding to unravel the subsurface reflection information from the data. We do not use *z*‐transforms and the associated Goupillaud's (1961) model, but use expressions in the time domain, with time denoted by *t*.

**Figure 1:**
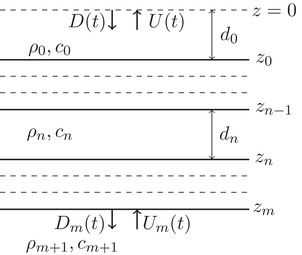
The model for a physical experiment in a one‐dimensional piecewise‐homogeneous medium with m+1 reflecting boundaries with transparent boundary at z=0, has down‐ and up‐going fields, D(t) and U(t) at z=0 and $D_{m}\left(t\right)$ and $U_{m}\left(t\right)$ at $z=z_{m}$, respectively.

Figure 1 shows the model configuration with possible up‐ and down‐going wave fields at the top and bottom of the layered medium. The up‐ and down‐going parts of the acoustic pressure, U(t) and D(t), respectively, are defined at z=0 and $U_{m}\left(t\right)$ and $D_{m}\left(t\right)$ just below $z=z_{m}$. The discrete‐layered medium has m+1 reflecting boundaries at depth levels $z_{n}$, n=0,1,…,m. Each layer is characterized by constant velocity, $c_{n}$, and density, $\rho_{n}$. The depth axis points downwards, hence $z_{n}>z_{n-1}$. The thickness of each layer is $d_{n}=z_{n}-z_{n-1}$, for n=1,2,…,m. For an interface at $z_{n}$, the reflection coefficient for a plane pressure wave coming from above is indicated by $r_{n}=\left(Z_{n+1}-Z_{n}\right)/\left(Z_{n+1}+Z_{n}\right)$, with the impedance given by $Z_{n}=\rho_{n}c_{n}$. The vertical travel time in each layer is denoted $t_{n}=d_{n}/c_{n}$ for n=1,2,…,m. We define the travel time and distance to *z*_0_ as $t_{0}=d_{0}/c_{0}$ and $d_{0}=z_{0}$. Cumulative travel time from z=0 to $z_{n}$ is denoted $t_{0n}=t_{0}+t_{1}+\cdots +t_{n}$. The transmission coefficient is denoted $t_{n}^{\pm }=\left(1\pm r_{n}\right)$ and the cumulative transmission coefficient is $t_{0n}^{\pm }=t_{0}^{\pm }t_{1}^{\pm }\cdots t_{n}^{\pm }$, where the plus‐sign applies to transmission from z=0 to $z=z_{n}$ and the minus‐sign from $z_{n}$ to z=0.

### Reflection and transmission experiments

**Figure 2:**
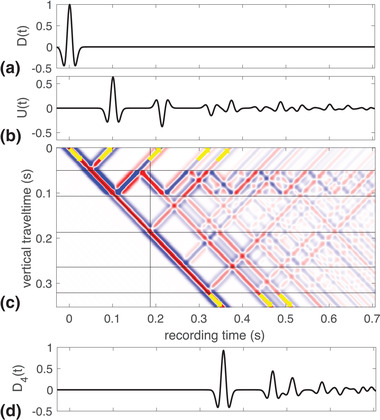
The down‐going (a) and up‐going (b) wave field at z=0, the wave field inside a medium with three reflectors as a function of vertical travel time and recording time (c) and the down‐going wave field below $z=z_{4}$ (d).

In the model shown in Fig. 1, the up‐ and down‐going fields are related to each other through the impulse reflection and transmission responses that is expressed as

(1) $$\left(\begin{array}{c} D_{m}\left(t\right)\\ U(t) \end{array}\right)=\left(\begin{array}{cc} T(t) & R(t)\\ R(t) & T(t) \end{array}\right)*\left(\begin{array}{c} D(t)\\ U_{m}\left(t\right) \end{array}\right),$$

where * denotes temporal convolution, R(t) and T(t) denote the impulse reflection and transmission responses, at z=0 and $z=z_{m}$, respectively, in case the down‐going wave field D(t)=δ(t) at z=0 and the up‐going field $U_{m}\left(t\right)=0$ at $z=z_{m}$, whereas R(t) and T(t) denote the impulse reflection and transmission responses, at $z=z_{m}$ and z=0, respectively, when the up‐going wave field $U_{m}\left(t\right)=\delta \left(t\right)$ just below $z=z_{m}$ and the down‐going field D(t)=0 at z=0. They are impulse responses, or Green's functions. The column vector in the left‐hand side of equation (1) is the down‐going wave field $D_{m}\left(t\right)$ that could be measured at $z=z_{m}$ and the up‐going wave field, U(t), that could be measured at z=0. The column vector in the right‐hand side of the equation contains the initiating down‐going wave field D(t) that could be emitted at z=0 and up‐going wave field $U_{m}\left(t\right)$ that could be emitted at $z=z_{m}$. These wave fields are shown in Fig. 1 and are connected to each other through the impulse reflection and transmission response matrix as expressed in equation (1). This is the convolutional model of a physical acoustic experiment. In this paper, all equations that represent an experiment have this structure. We illustrate equation (1) with a numerical example. We take a model with five reflectors (m=4), which we use throughout the paper. The medium parameters and the local reflection coefficients are given in Table 1. We emit a Ricker wavelet at 75 m above the top reflecting boundary. It is shown as the down‐going field, D(t), in Fig. 2(a) and $U_{4}\left(t\right)=0$. The reflection response, U(t), is shown in Fig. 2(b). The total wave field everywhere in the model is shown in Fig. 2(c) as a function of vertical travel time, ζ, and recording time, *t*. The reflectors are indicated by the horizontal black lines and the vertical black line indicates the time it takes the initial down‐going wave to propagate to *z*_2_. We use this level later to introduce a truncated medium which facilitates our analysis. The slanted black line marks ζ=t. Zero vertical travel time indicates the acquisition surface in the top. The down‐going field $D_{4}\left(t\right)$ is shown in Fig. 2(d). The sum of the two traces in Fig. 2(a,b) is the same as the field at zero vertical travel time in Fig. 2(c). The wave field in Fig. 2(d) is the same as the field shown at the latest vertical travel time in Fig. 2(c). Some of these events are indicated in Fig. 2(c) with arrows to show in which direction they propagate.

**Table 1:** Values for velocity, density, layer thickness and local reflection coefficient in the layered model

Velocity (m/s)	Density (kg/m^3^)	Thickness (m)	reflection Coefficient (−)
1500	1000	∞	0.6364
3000	2250	117	−0.6364
1500	1000	99	0.4545
2000	2000	85	−0.2075
1750	1500	111	0.3538
2750	2000	∞	–

### Time‐reversed reflection and transmission experiments

**Figure 3:**
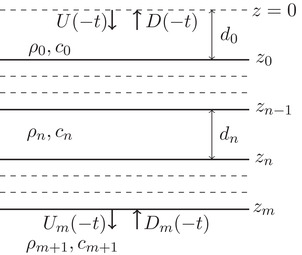
The time‐reversed experiment that corresponds to the physical experiment of Fig. 1 has down‐ and up‐going fields, U(−t) and D(−t) at z=0, and $U_{m}\left(-t\right)$ and $D_{m}\left(-t\right)$ at $z=z_{m}$, respectively.

In a lossless medium, the wave equation is symmetric in time, which we exploit in a time‐reversed experiment. A down‐going field at the acquisition surface in a physical experiment is time reversed and becomes an up‐going field in a time‐reversed experiment and an up‐going field becomes a down‐going field upon time reversal. This configuration is shown in Fig. 3. By carrying out a time‐reversed experiment using the responses measured in the physical experiment, we recover our original source time function as the response (Fink 1992). Interchanging the up‐ and down‐going fields in the column vectors of equation (1) and reversing their time dependency gives the expression for a time‐reversed experiment, which is then given by

(2) $$\left(\begin{array}{c} U_{m}\left(-t\right)\\ D(-t) \end{array}\right)=\left(\begin{array}{cc} T(t) & R(t)\\ R(t) & T(t) \end{array}\right)*\left(\begin{array}{c} U(-t)\\ D_{m}\left(-t\right) \end{array}\right).$$

Equation (2) is the mathematical expression of the experiment depicted in Fig. 3. For later convenience, we take the time reverse of equation (2), reorder the fields to make an equation that resembles equation (1) and find

(3) $$\left(\begin{array}{c} D(t)\\ U_{m}\left(t\right) \end{array}\right)=\left(\begin{array}{cc} T(-t) & R(-t)\\ R(-t) & T(-t) \end{array}\right)*\left(\begin{array}{c} D_{m}\left(t\right)\\ U(t) \end{array}\right).$$

From here onward, equation (3) is how we define a time‐reversed experiment. It shows that when the field responses, U(t) and $D_{m}\left(t\right)$, at z=0 and $z=z_{m}$, respectively, generated by D(t) and $U_{m}\left(t\right)$ in the physical experiment, are used as the emitted wave fields in a time‐reversed experiment, the response is the original emitted field D(t) and $U_{m}\left(t\right)$. This is the convolutional model of the time‐reversed experiment corresponding to that of the physical experiment. We interpret Fig. 2 using equation (3). Equation (3) shows in the column vector in the right‐hand side that the wave fields shown in Fig. 2(b,d) are the input wave fields, U(t) from above and $D_{m}\left(t\right)$ from below, respectively. According to equation (3), the medium responds in reverse time. This means that the wave field propagates inside the layered medium in the direction of decreasing recording time. This can be seen in Fig. 2(c) when all arrows are rotated 180°, such that what was upward pointing is now downward pointing, and vice versa, and they all point towards decreasing recording time. Consequently, the only result is the Ricker wavelet shown in Fig. 2(a).

We use the fact that the input fields ${\left(D_{m}\left(t\right),U\left(t\right)\right)}^{t}$, where superscript *t* denotes matrix transposition, in equation (3), are the responses in the left‐hand side of equation (1). Combining these two equations results in a statement that the input is the same as the output. Since this must be true for any input, we decide to use once D(t)=δ(t) and $U_{m}\left(t\right)=0$ and once D(t)=0 and $U_{m}\left(t\right)=\delta \left(t\right)$, which results in the matrix expression

(4) $$\left(\begin{array}{cc} \delta (t) & 0\\ 0 & \delta (t) \end{array}\right)=\left(\begin{array}{cc} T(-t) & R(-t)\\ R(-t) & T(-t) \end{array}\right)*\left(\begin{array}{cc} T(t) & R(t)\\ R(t) & T(t) \end{array}\right),$$

which expresses the unitary property of the reflection and transmission impulse response matrix. It is the well‐known expression of the conservation of acoustic energy in the system. The two equations involving the first column of the second matrix in the right‐hand side of equation (4) are written as

(5) $$\begin{array}{c} \delta (t)=R(-t)*R(t)+T(-t)*T(t), \end{array}$$

(6) $$\begin{array}{c} \;0=T(-t)*R(t)+R(-t)*T(t). \end{array}$$

Equations (5) and (6) describe the time‐reverse experiment of equation (3), with the incident fields given by U(t)=R(t) and $D_{m}\left(t\right)=T\left(t\right)$. Equation (5) was given in Robinson and Treitel (1977) and forms the basis of their methods, which we discuss later. We use these relations when we want to interrogate the medium in its interior using only the reflection response R(t).

### Relations between the reflection and transmission responses and the fundamental wave field

Impulse reflection and transmission responses are not independent from each other. Compact frequency domain expressions were given in terms of the up‐ and down‐going parts of a fundamental wave field in optics by Abeles (1946) and in acoustics by Goupillaud (1961). Kunetz and d'Erceville (1962) introduced the term fundamental polynomials and Kunetz (1964) gave coupled recurrence relations for the fundamental polynomials. Here we give a treatment in our notation to link their equations to the recent work on Marchenko‐type equations to obtain reflectivity from the reflection response (Slob *et al*. 2014). If a layered system has n+1 reflecting boundaries, with the bottom reflector at $z_{n}$ and n<m, we call it a truncated medium. Its impulse reflection response from a source in the top is denoted $R_{n}$ and its impulse transmission response just below $z_{n}$ is denoted $T_{n}$. They are given in terms of the two parts of the fundamental wave field related to the truncated medium, $h_{n}^{-}\left(t\right)$ and $h_{n}^{+}\left(t\right)$, as

(7) $$\begin{array}{c} \;h_{n}^{-}\left(t\right)=R_{n}\left(t\right)*h_{n}^{+}\left(t\right), \end{array}$$

(8) $$\begin{array}{c} T_{d,n}\left(t\right)=t_{0n}^{+}\delta \left(t-t_{0n}\right)=T_{n}\left(t\right)*h_{n}^{+}\left(t\right), \end{array}$$

where $t_{0n}^{+}$ denotes the cumulative transmission coefficient, as defined in the paragraph describing Fig. 1, and $T_{d,n}\left(t\right)$ is explained below. Equations (7) and (8) can be interpreted as the convolutional model of a seismic reflection and transmission experiment. According to equation (1), when $D\left(t\right)=h_{n}^{+}\left(t\right)$ is the down‐going wave field incident on the truncated medium and $U_{n}\left(t\right)=0$, $U\left(t\right)=h_{n}^{-}\left(t\right)$ is the reflection response and $D_{n}\left(t\right)=t_{0n}^{+}\delta \left(t-t_{0n}\right)$ the transmission response. The latter is the physical direct arrival in the impulse transmission response, which we denote as $T_{d,n}\left(t\right)$ as shown in equation (8). For this reason, we interpret $h_{n}^{+}\left(t\right)$ as the internal multiple eliminator, or the anti‐reverberation filter, for the transmission response of the truncated medium. The expression of equation (7) was given in Kunetz (1964).

The special character of the two wave fields $h_{n}^{\pm }$ is captured in their coupled recurrence relations given by (Kunetz 1964)

(9) $$\begin{array}{c} h_{n+1}^{+}\left(t\right)=h_{n}^{+}\left(t\right)+r_{n+1}h_{n}^{-}\left(2t_{0(n+1)}-t\right), \end{array}$$

(10) $$\begin{array}{c} h_{n+1}^{-}\left(t\right)=h_{n}^{-}\left(t\right)+r_{n+1}h_{n}^{+}\left(2t_{0(n+1)}-t\right), \end{array}$$

where $h_{n+1}^{\pm }$ are the up‐ and down‐going parts of the fundamental wave field for the truncated medium that has its bottom reflector at $z_{n+1}$. Both equations are used in the method of Kunetz (1964). The forward recursion is initialized by

(11) $$\begin{array}{c} h_{0}^{+}\left(t\right)=\delta \left(t\right), \end{array}$$

(12) $$\begin{array}{c} h_{0}^{-}\left(t\right)=r_{0}\delta \left(t-2t_{0}\right). \end{array}$$

The importance of the initial unit amplitude impulse lies in the fact that $h_{n}^{+}$ is a causal minimum delay function for arbitrary *n*, provided all $|r_{n}|<1$. For this reason, the inverse exists, denoted $M_{n}\left(t\right)$, which is a causal minimum delay function as well, with $M_{n}\left(t\right)*h_{n}^{+}\left(t\right)=\delta \left(t\right)$. Because $h_{n}^{+}\left(t\right)$ is understood as the multiple eliminator, $M_{n}\left(t\right)$ is understood as the multiple generator, such that $R_{n}=h_{n}^{-}\left(t\right)*M_{n}\left(t\right)$.

It is of interest to make three observations from equations (9) and (10). First, $h_{n}^{\pm }$ have the same finite number of events and their number is equal to 2^*n*^. This can be understood from the fact that for n=0 both filters have one event, see equations (11) and (12), and every time we add a reflector, we double the number of events. Second, all primary reflections that occur in the impulse reflection response have the local reflection coefficient as amplitude in $h_{n}^{-}\left(t\right)$ and arrive at the expected two‐way travel time. This can be seen from equation (10) as follows. Let us take n=0, $h_{1}^{-}\left(t\right)$ is then constructed from $h_{0}^{-}\left(t\right)$, which contains the first reflection event at its physical arrival time, and which will never change when we increase *n*. The second term in the right‐hand side has a unit amplitude impulse in $h_{0}^{+}\left(t\right)$, which results in the contribution $r_{1}\delta \left(t-2t_{01}\right)$ and contains the local reflection coefficient as an event at the physical arrival time. Then we take n=1 and $h_{2}^{-}\left(t\right)$ has the reflection events from the first two reflectors as present in $h_{1}^{-}\left(t\right)$ and $r_{2}\delta \left(t-2t_{02}\right)$ is obtained from the unit amplitude impulse in $h_{1}^{+}\left(t\right)$. The second event in $h_{1}^{+}\left(t\right)$ will create a non‐physical event in $h_{2}^{-}\left(t\right)$. This will happen for any value of *n* where all coefficients $r_{k}$, k=0,1,…,n, are contained in $h_{n}^{-}\left(t\right)$ and $r_{n+1}\delta \left(t-2t_{0(n+1)}\right)$ comes from the leading impulse in $h_{n}^{+}\left(t\right)$. Third, in $h_{n}^{\pm }$ no events are present outside the time window between t=0 and $t=2t_{0n}$. All events in $h_{n}^{-}$ are primary reflections and the ones that can be traced back to the initial unit amplitude impulse at t=0 are the physical primary reflections. Emitting $h_{n}^{+}$ into the truncated medium leads to the minimum number of events possible in a reflection and transmission experiment for that truncated medium. The reflection response is $h_{n}^{-}$, which has the same number of events as the input signal. In this sense, the functions $h_{n}^{\pm }$ are called the up‐ and down‐going parts of the fundamental wave field, $h_{n}=h_{n}^{+}+h_{n}^{-}$. The transmission response has a single event, coming from the initial down‐going impulse in $h_{n}^{+}$.

**Figure 4:**
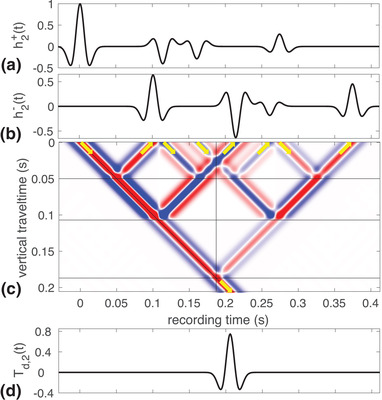
The down‐going anti‐reverberation filter (a) and up‐going truncated medium response (b) at z=0, the wave field inside the truncated medium with three reflectors as a function of vertical travel time and recording time (c) and the down‐going transmission response of the truncated medium in the bottom below $z=z_{2}$ (d).

To illustrate this, we use a truncated part of the model as in Fig. 2, with n=2. We show the wave fields in the truncated medium in Fig. 4. The down‐going field, $h_{2}^{+}\left(t\right)$, is shown in Fig. 4(a). The corresponding reflection response, $h_{2}^{-}\left(t\right)$, is shown in Fig. 4(b). How the waves in Fig. 4(a) propagate into the medium and lead to waves in the response shown in Fig. 4(b) is shown in Fig. 4(c). Similar to Fig. 2(c), here the plot shows the propagation throughout the medium as a function of vertical travel time, ζ, and recording time, *t*. The reflectors are indicated by the horizontal black lines and the vertical black line indicates the time it takes the initial down‐going wave to propagate to *z*_2_. The slanted black line marks ζ=t. Zero vertical travel time indicates the acquisition surface. In the figure, the four waves that are emitted into the medium are indicated by the downward pointing arrows. These are the same as the ones shown in Fig. 4(a). The four waves that arrive at the acquisition surface are indicated by the upward pointing arrows. These are the same as the ones shown in Fig. 4(b). Note that the acquisition surface is an acoustic transparent surface where no reflections occur. Just below the black line that marks the bottom reflector we see the single wave propagating down into the lower half space as expressed in the left‐hand side of equation (8) with n=2 and indicated by the downward pointing arrow in the bottom of the figure. This is the physical first arrival in an impulse transmission experiment and is the only down‐going wave as shown in Fig. 4(d). This confirms that $h_{2}^{+}\left(t\right)$ is the anti‐reverberation filter for the transmission response of the truncated medium. It has the same meaning for the reflection response, albeit that in that case non‐physical primary reflections end up in $h_{2}^{-}\left(t\right)$ as can be seen in Fig. 4(b) where the third event is a non‐physical primary reflection. This event arises from the third event in $h_{2}^{+}\left(t\right)$ which is emitted to prevent a multiple to be generated at the second reflector. Only then a single wave travels down below the third reflector.

To understand the corresponding time‐reversed experiment, we replace the reflection and transmission responses in equations (5) and (6) by those of the truncated medium. We then convolve both sides by $h_{n}^{+}$ and use equations (7) and (8) to find

(13) $$\begin{array}{c} h_{n}^{+}\left(t\right)=R_{n}\left(-t\right)*h_{n}^{-}\left(t\right)+T_{n}\left(-t\right)*T_{d,n}\left(t\right), \end{array}$$

(14) $$\begin{array}{c} \;0=T_{n}\left(-t\right)*h_{n}^{-}\left(t\right)+R_{n}\left(-t\right)*T_{d,n}\left(t\right), \end{array}$$

where $R_{n}$ and $T_{n}$ are defined as R and T but now for the truncated medium. Let us interpret Fig. 4 as a time‐reversed experiment. By rotating all arrows 180° in Fig. 4(c), all propagation takes place in the reversed‐time direction. The waves shown in Fig. 4(b) are now sent into the medium at zero vertical travel time and propagate in the direction of decreasing recording time. The reflection response at z=0 is given by the first term in the right‐hand side of equation (13). The corresponding transmission response at $z=z_{2}$ is given by the first term in the right‐hand side of equation (14). The wave in the bottom of the model is now an up‐going wave incident on the three reflectors and it propagates through the model in the decreasing time direction. Its initial strength is $t_{02}^{+}$ and it starts at $t=t_{02}$. The transmission response at z=0 is given by the second term in the right‐hand side of equation (13). The corresponding reflection response from below at $z=z_{2}$ is given by the second term in the right‐hand side of equation (14). The sum of the two terms in the right‐hand side of equation (13) forms the total response at the acquisition surface, z=0, given by the left‐hand side of equation (13). These are the waves shown in Fig. 4(a). The sum of the two terms in the right‐hand side of equation (14) forms the total response just below $z=z_{2}$. This total response is zero as can be seen in Fig. 4(c), where no waves exist below the bottom reflector as a function of decreasing recording time and increasing vertical travel time. Hence, the waves in Fig. 4(c) can be understood as propagating in increasing or decreasing recording time direction without changing anything in their amplitude and time behaviour.

If we convolve both sides of equation (13) with $h_{n}^{+}\left(-t\right)$, use equation (7) and reorder the terms, we obtain the useful relation (Kunetz 1964)

(15) $$h_{n}^{+}\left(t\right)*h_{n}^{+}\left(-t\right)-h_{n}^{-}\left(t\right)*h_{n}^{-}\left(-t\right)=t_{0n}^{+}t_{0n}^{-}\delta \left(t\right).$$

To obtain the right‐hand side of equation (15), we have used an equation similar to equation (8) but for transmission from bottom to top, with $T_{n}\left(t\right)$ replaced by $T_{n}\left(t\right)$ and $t_{0n}^{+}$ replaced by $t_{0n}^{-}$. We use equation (15) when we discuss inversion methods in the next section.

### Single‐sided time‐reversed experiment

**Figure 5:**
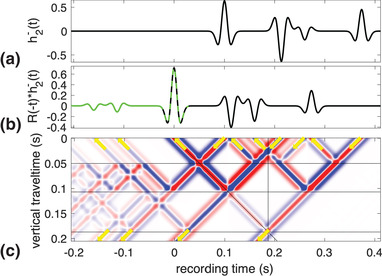
The time‐reversed filter responses in the truncated medium; (a) The incident wave field, $h_{2}^{-}\left(t\right)$; (b) the reflected wave field composed of $h_{2}^{+}\left(t\right)$ (black line), $-t_{02}^{+}G^{-}\left(0,z_{2},t_{02}-t\right)$ (green line) and the sum where they overlap (dashed black‐green) at z=0; (c) the wave field propagating in reverse time inside a medium with three reflectors as a function of vertical travel time and recording time.

The above time‐reversed experiments were carried out as two‐sided experiments. We want to be able to find the medium parameters using only the single‐sided reflection response. For this reason, we assume that we know only the reflection response at z=0 and write equation (13) as

(16) $$\begin{array}{c} h_{n}^{+}\left(t\right)-G^{-}\left(0,z_{n},-t\right)*T_{d,n}\left(t\right)=R_{n}\left(-t\right)*h_{n}^{-}\left(t\right), \end{array}$$

where $G^{-}\left(0,z_{n},t\right)=T_{n}\left(t\right)$ is the Green's function describing the pressure at z=0 generated by an up‐going impulse just below $z=z_{n}$ as indicated by the minus‐sign in superscript. Equation (16) states that when the up‐going fundamental wave field is sent into the medium for a time‐reversed experiment, the reflection response is the down‐going fundamental wave field minus the time‐reversed Green's function convolved with the direct arrival in the transmission response. Equation (16) is of course the same equation as equation (13), but with different meaning and interpretation. We illustrate equation (16) as a single‐sided time‐reversed experiment in Fig. 5. In this situation, the field that is incident on the medium only comes from above and is $h_{2}^{-}\left(t\right)$, shown in Fig. 5(a). The time‐reversed reflection response of the medium is shown in Fig. 5(b) where the function $h_{2}^{+}\left(t\right)$ minus the scaled and delayed Green's function are depicted. The part of the filter that does not overlap with minus the scaled and delayed Green's function is shown in black, whereas the part of minus the scaled and delayed Green's function that does not overlap with the filter is shown in green. It can be seen that the two functions have one overlapping event, which is depicted as a dashed black–green line, because it is the sum of the two terms. The difference between Figs. 4(c), interpreted as a time‐reversed experiment, and 5(c) is the up‐going incident wave from the bottom that is present in Fig. 4(c) and absent in 5(c). This absence can be understood as emitting that impulse together with the same impulse with opposite sign. Consequently, no up‐going wave is visible in the bottom of Fig. 5(c) and as a result there are propagating waves that arise from the extra negative amplitude impulse that leads to minus the scaled and delayed time‐reversed Green's function at the acquisition surface. With this understanding, we are ready to use the fundamental wave field of the truncated medium in physical and time‐reversed experiments using the impulse reflection response of the actual medium.

### Filtering the impulse reflection response with fundamental wave fields of a truncated medium

In this section, we derive expressions for physical and time‐reversed experiments when the incident field is coming from above only. We begin with writing the impulse reflection response R(t) as the sum of the impulse reflection response of the truncated medium and a scaled and delayed Green's function. This was derived by Goupillaud (1961) and we write his expression as

(17) $$R\left(t\right)=R_{n}\left(t\right)+G^{+}\left(0,z_{n},t\right)*T_{n}\left(t\right),$$

where $G^{+}\left(0,z_{n},t\right)$ is a Green's function of the actual layered medium. It describes the acoustic pressure at z=0, generated by a down‐going unit‐amplitude impulse just below $z_{n}$ as indicated by the plus‐sign in superscript. The convolution of $T_{n}$ and $G^{+}$ describes the part of the impulse reflection response that is not described by $R_{n}$. We convolve all terms in equation (17) with $h_{n}^{+}\left(t\right)$, use equations (7) and (8) and write it as a single‐sided experiment given by

(18) $$h_{n}^{-}\left(t\right)+G^{+}\left(0,z_{n},t\right)*T_{d,n}\left(t\right)=R\left(t\right)*h_{n}^{+}\left(t\right).$$

**Figure 6:**
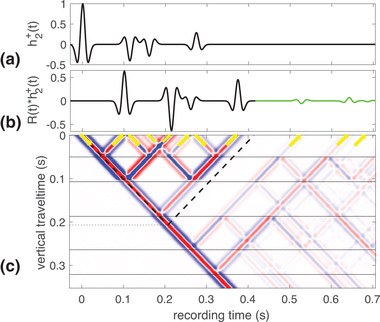
The response of the actual medium to $h_{2}^{+}\left(t\right)$; (a) incident field $h_{2}^{+}\left(t\right)$, (b) reflection response composed of $h_{2}^{-}\left(t\right)$ (black line) and $t_{02}^{+}G^{+}\left(0,z_{2},t-t_{02}\right)$ (green line) at z=0 and (c) the wave field propagating in a medium with five reflectors as a function of vertical travel time and recording time.

Equation (18) states that when the incident field is the down‐going part of the fundamental wave field of the truncated medium, the reflection response is the up‐going part of the fundamental wave field of the truncated medium plus a scaled and delayed Green's function of the actual medium. There is no overlap in time between these two parts of the reflection response. To show that, we illustrate equation (18) with a numerical example with m=4 and n=2. Figure 6(a) shows $h_{2}^{+}\left(t\right)$ as incident field which is the same as in Fig. 4(a). Figure 6(b) shows the corresponding reflection response, the black line is $h_{2}^{-}\left(t\right)$ and is the same as in Fig. 4(b), whereas the green line is the scaled and delayed Green's function. Figure 6(c) shows the wave field in the actual medium. The dotted line marks the vertical travel time where the actual medium is truncated. The black solid lines indicate the reflecting boundaries and the dashed lines indicate the time window of the fundamental wave fields. This means that the part inside the time window marked by the dashed lines is the same as Fig. 4(c). Consequently, all waves outside the time window marked by the dashed lines are part of the delayed and scaled Green's function. As long as we truncate the actual medium between the third and fourth reflector, the wave field does not change anywhere.

Equation (18) was used by Kunetz to invert a trace with a forward recursion method using equations (9) and (10). Equation (18) is also one of the two equations used for the Marchenko method that makes non‐recursive use possible. Before we can demonstrate that, we must obtain an equation for the single‐sided time‐reversed experiment. We need the expression for the impulse transmission response, which is written as

(19) $$T\left(t\right)=G^{+}\left(z_{m},z_{n},t\right)*T_{n}\left(t\right),$$

where $G^{+}\left(z_{m},z_{n},t\right)$ is the pressure Green's function of the actual layered medium. It describes the impulse response just below $z_{m}$ generated by the unit amplitude down‐going impulse starting just below $z_{n}$. When the actual impulse is not just below $z_{n}$ but at z=0, we need to convolve the Green's function with the transmission response of the truncated medium. This follows directly from the convolutional model of an acoustic experiment. We write equation (19) with the aid of equation (8) as an experiment, given by

(20) $$G^{+}\left(z_{m},z_{n},t\right)*T_{d,n}\left(t\right)=T\left(t\right)*h_{n}^{+}\left(t\right).$$

The left‐hand side of equation (20) is the wave field just below the black line marking the bottom reflector in Fig. 6(c).

In the two‐sided time‐reversed experiment, the responses as given in the left‐hand sides of equations (18) and (20) are emitted into the medium from top and bottom, respectively, and propagation takes place in reversed time. This leads to $h_{n}^{+}$ as the response at the acquisition surface, given by

(21) $$\begin{array}{ccc} h_{n}^{+}\left(t\right) & = & R\left(-t\right)*\left[h_{n}^{-}+G^{+}\left(0,z_{n},t\right)*T_{d,n}\left(t\right)\right]\\ & & +\;T\left(-t\right)*\left[G^{+}\left(z_{m},z_{n},t\right)*T_{d,n}\left(t\right)\right]. \end{array}$$

Equation (21) is found by convolving all terms in equation (5) with $h_{n}^{+}\left(t\right)$ and then using equations (18) and (20) in the resulting right‐hand side. We understand equation (21) from Fig. 6(c) in the way we used Fig. 4(c) to understand equation (13). In a time‐reversed experiment, there are many waves sent in from below and from above by the scaled and delayed Green's functions, together with the waves in $h_{2}^{-}$ that are sent in from above. They result in $h_{2}^{+}$ at the acquisition surface. Sending in the scaled and delayed Green's functions from the top and bottom leads to a single up‐going impulse with amplitude $t_{02}^{+}$ that arrives just below *z*_2_ at $t=t_{02}$. It will then continue to propagate in reverse time. At the acquisition surface, this would lead to the time‐reversed scaled and delayed Green's function $t_{02}^{+}G^{-}\left(0,z_{2},t_{02}-t\right)$. Just below *z*_4_, it would be the time‐reversed scaled and delayed Green's function $t_{02}^{+}G^{-}\left(z_{4},z_{2},t_{02}-t\right)$. However, because we send $h_{2}^{-}$ back into the medium as well, these scaled and delayed time‐reverse Green's functions are cancelled. Hence, no waves exist for recording times smaller than the vertical travel time as can be seen in Fig. 6(c). The sum of the two terms involving the Green's functions in the right‐hand side of equation (21) is equal to the scaled and delayed time‐reversed impulse response, or the convolution of the Green's function with the direct arrival of the transmission response in the truncated medium, hence $G^{-}\left(0,z_{2},-t\right)*T_{d,2}\left(t\right)=t_{02}^{+}G^{-}\left(0,z_{2},t_{02}-t\right)$, at the acquisition surface. This is true for any *n* and *m* with n=2 and m=4 being an arbitrary example.

We carry out a single‐sided time‐reversed experiment and send in only $h_{n}^{-}\left(t\right)$. In that case, this time‐reversed Green's function will be part of the response with a minus sign. For this reason, we rewrite equation (21) as

(22) $$h_{n}^{+}\left(t\right)-G^{-}\left(0,z_{n},-t\right)*T_{d,n}\left(t\right)=R\left(-t\right)*h_{n}^{-}\left(t\right),$$

with

(23) $$\begin{array}{ccc} G^{-}\left(0,z_{n},-t\right) & = & R\left(-t\right)*G^{+}\left(0,z_{n},t\right)\\ & & +\;T\left(-t\right)*G^{+}\left(z_{m},z_{n},t\right). \end{array}$$

**Figure 7:**
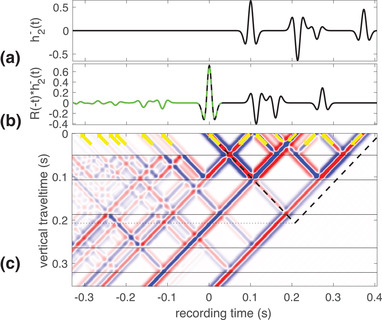
The time‐reversed response of the actual medium to $h_{2}^{-}\left(t\right)$;(a) the incident wave field, $h_{2}^{-}\left(t\right)$; (b) the reflected wave field composed of $h_{2}^{+}\left(t\right)$ (black line) and $t_{02}^{+}G^{-}\left(0,z_{2},t_{02}-t\right)$ (green line) at z=0 of a time‐reversed experiment; (c) the wave field propagating in reverse time inside a medium with five reflectors as a function of vertical travel time and recording time.

Equation (22) is interpreted in the same way as equation (16). The only difference is that now the Green's function belongs to the actual medium and not to the truncated medium. We illustrate equation (22) with a numerical example where the waves propagate in the same model as used for Fig. 6, but now in reversed time. We send in only $h_{2}^{-}\left(t\right)$ and the reflection response is as given in the left‐hand side of equation (22). Equation (22) is of course the same as equation (21) but interpreted differently. The wave field corresponding to the interpretation of equation (22) is shown in Fig. 7. Figure 7(a) shows the emitted wave field $h_{2}^{-}\left(t\right)$ and Fig. 7(b) shows the corresponding reflection response, which again consists of two terms that overlap at t=0 and their sum is shown as a dashed green–black line. The rest of $h_{2}^{+}\left(t\right)$ is shown in black, whereas the rest of minus the scaled and delayed time‐reversed Green's function is shown in green. Figure 7(c) shows the waves in the entire medium and the arrows indicate the direction of propagation at the acquisition surface. Similar to the missing up‐going wave in the bottom of Fig. 5(c), here the up‐going wave where the dashed and dotted lines coincide is absent. Therefore, similar to what we saw in Fig. 5(b), there is an overlap at zero time of the two terms in the left‐hand side of equation (22), but otherwise the two parts of the reflection response are disjoint.

To facilitate the derivation of the Marchenko equation and later analysis of non‐recursive inversion, we change the notation of the fundamental wave fields to allow positioning the down‐going impulse at an arbitrary vertical travel time ζ. We write them as $h^{\pm }\left(0,\zeta ,t\right)$ with ζ as a free parameter that defines the time window in which the function exists. Hence, $h^{\pm }\left(0,\zeta ,t\right)=0$ for t<0 and t>2ζ and $h^{\pm }\left(0,\zeta ,t\right)=h_{n}^{\pm }\left(t\right)$ for $t_{0n}<\zeta <t_{0(n+1)}$. With this new notation, we write equations (18) and (22) in the time domain as

(24) $$\begin{array}{c} h^{-}\left(0,\zeta ,t\right)+t^{+}\left(\zeta \right)G^{+}\left(0,\zeta ,t-\zeta \right)=R\left(t\right)*h^{+}\left(0,\zeta ,t\right), \end{array}$$

(25) $$\begin{array}{c} h^{+}\left(0,\zeta ,t\right)-t^{+}\left(\zeta \right)G^{-}\left(0,\zeta ,\zeta -t\right)=R\left(-t\right)*h^{-}\left(0,\zeta ,t\right) \end{array}$$

where $t^{+}\left(\zeta \right)=t_{0n}^{+}$ for $t_{0n}<\zeta <t_{0(n+1)}$.

### Derivation of the Marchenko equation

For the later purpose of Marchenko inversion, we derive the Marchenko equation. We define a shifted version of the fundamental wave field as $k^{\pm }\left(0,\zeta ,t\right)=h^{\pm }\left(0,\zeta ,t+\zeta \right)$ for −ζ<t<ζ, where it is noted that the impulse at zero time, which is the first term in $h^{+}\left(0,\zeta ,t\right)$, is not included in $k^{+}\left(0,\zeta ,t\right)$. We use this wave field in equations (24) and (25), take the time‐reverse of the latter equation and restrict the time window such that the contributions from the Green's functions are excluded. This means we evaluate the equations in the interval −ζ<t<ζ and find

(26) $$\begin{array}{c} R\left(t\right)*k^{+}\left(0,\zeta ,t\right)+R\left(t+\zeta \right)-k^{-}\left(0,\zeta ,t\right)=0, \end{array}$$

(27) $$\begin{array}{cc} & \;R\left(t\right)*k^{-}\left(0,\zeta ,-t\right)-k^{+}\left(0,\zeta ,-t\right)=0, \end{array}$$

where R(t+ζ) shows up as a separate term in the left‐hand side of equation (26), because the initial delta‐function of $h^{+}\left(0,\zeta ,t\right)$ is not part of the function $k^{+}\left(0,\zeta ,t\right)$ but it does contribute to the convolution in equation (24). We are free to add or subtract the two equations and decide to subtract. We introduce the fundamental wave field $k\left(0,\zeta ,t\right)=k^{+}\left(0,\zeta ,t\right)-k^{-}\left(0,\zeta ,-t\right)$ and end up with

(28) $$\int_{t^{\prime }=-\zeta }^{t}R\left(t-t^{\prime }\right)k\left(0,\zeta ,t^{\prime }\right)dt^{\prime }+k\left(0,\zeta ,-t\right)+R\left(t+\zeta \right)=0,$$

and −ζ<t<ζ. Equation (28) is the Marchenko equation. The functions $k^{\pm }\left(0,\zeta ,t\right)$ are the same as the functions $h^{\pm }\left(z_{0},z_{i},t\right)$ as defined in equations (21) and (22) in Slob *et al*. (2014), but they used *z*_0_ instead of z=0 and used a depth level $z_{i}$ instead of vertical travel time ζ. With this result, the relation between the fundamental wave fields and the kernel of the Marchenko equation is established.

## FOUR INVERSION ALGORITHMS

In this section, we describe the inverse filtering method of Kunetz (1964) who used a forward recursive scheme and of Robinson and Treitel (1978) who used a backward recursive scheme. We then use the Marchenko equation to directly compute the impedance for any vertical travel time ζ as given in Berryman and Greene (1980). We end the section with the non‐recursive scheme to obtain the local reflection coefficient at any vertical travel time. All four methods presented here are exact for infinite bandwidth data. As we show below, the direct Marchenko inversion computes the impedance from the zero‐frequency value in the data, which normally is not available. The other three methods offer a data filtering technique that can be used when the source wavelet is known from pre‐processing, because they do not rely on the zero‐frequency information to be present in the data. Presence of the source time signature in the data brings band‐limitation and the associated limited resolution in the proper retrieval of reflection coefficients. In one‐dimension (1D), the information available and retrievable from the reflection response is at best the acoustic impedance as a function of vertical travel time. All four schemes perform this task through direct data filtering methods. In this sense, full waveform inversion using only the subsurface reflection response of a discretely layered lossless 1D medium is a linear problem. The presence of the pressure‐free or rigid surface as acquisition surface does not increase the number of unknowns and slightly modified versions of the schemes presented here will remain valid and exact. Kunetz (1964) and Robinson and Treitel (1978) have included those surfaces in their analysis. Singh *et al*. (2015) has included it for the Marchenko scheme. It follows from the analysis in Zhang and Slob (2019) for the Marchenko‐type scheme.

### Kunetz' inversion method by forward recursion

Kunetz and d'Erceville (1962) derived the recursive expressions for the fundamental wave field and included the effects of a pressure‐free or rigid surface. Kunetz (1964) recognized that the fundamental wave field can be used in a recursive manner to remove overlap from shallow multiples from the first primary that occurs below that shallow part. His algorithm includes the free surface, but here we assume it is not present. The reasoning is as follows. Because it is a forward recursive scheme, we compute filters from previous inversion results and find the reflection coefficient from the next primary in the data. Hence, all reflection coefficients are obtained from the data, not from the filter. The first reflection in the data is a primary with the local reflection coefficient as amplitude, hence *r*_0_ is found directly from the first event in R(t) and we find it as

(29) $$r_{0}\delta \left(t-2t_{0}\right)=R\left(t\right)\left[1-H\left(t-2t_{0}-\varepsilon \right)\right],$$

where ε is a small number, but large enough to ensure that the reflection occurs in the non‐zero part of the windowed reflection response as expressed in the right‐hand side of the equation. In practice, half the time length of the source wavelet is used. We then know *r*_0_ and *t*_0_ with which the associated fundamental wave field is computed according to equations (11) and (12). We also know $t_{0}^{\pm }$ and perform the following recursive steps for n≥1. We compute the next reflection coefficient from equation (18) as

(30) $$\begin{array}{ccc} & & r_{n}\delta \left(t-2t_{0n}\right)\\ & & \;=\frac{\left[R\left(t\right)*h_{n-1}^{+}\left(t\right)\right]\left[H\left(t-T_{0n}^{-}\right)-H\left(t-T_{0n}^{+}\right)\right]}{t_{0(n-1)}^{+}t_{0(n-1)}^{-}}. \end{array}$$

where $T_{0n}^{\pm }=2t_{0n}\pm \varepsilon$. Note that in the right‐hand side of equation (30) we convolve the data with $h_{n-1}^{+}\left(t\right)$, while the truncation is around $2t_{0n}$. The convolution with $h_{n-1}^{+}\left(t\right)$ removes all multiples generated at any of the reflectors from the surface to $z_{n-1}$ from the data. In the convolution result, the first event after $t=2t_{0(n-1)}$ is the primary reflection from the reflector at $z_{n}$ and this is the first event in the scaled and delayed Green's function in equation (18). This has the cumulative two‐way transmission coefficients in its amplitude and that is why this factor is present in the denominator of equation (30). Then $h_{n}^{\pm }\left(t\right)$ is computed from equations (9) and (10), we add 1 to *n* and evaluate equation (30) again until the entire trace is predicted. Note that the denominator in the right‐hand side of equation (30) can be found directly using equation (15). The impedance of each layer is found from

(31) $$Z_{n+1}=Z_{n}\frac{1+r_{n}}{1-r_{n}},$$

and this completes the inversion.

Based on this same idea, a slightly different version of the forward recursive scheme was implemented in Robinson (1967) and was derived in detail in Robinson and Treitel (1977) who also gave a numerical example. Kunetz (1964) showed that the acoustic response at depth, generated by an impulsive source at the free surface, is a function whose autocorrelation has a causal part that is the reflection response that would be measured at the surface. By reciprocity, as was already remarked by Robinson and Treitel (1977), this is the same as what Claerbout (1968) wrote.

### Robinson and Treitel's inversion method by backward recursion

Robinson and Treitel (1978) recognized that all local reflection coefficients are present at their correct two‐way travel time in the up‐going part of the fundamental wave field computed for the bottom reflector, $h_{m}^{-}\left(t\right)$. They write that if higher order products of reflection coefficients can be neglected, we need to solve only for $h_{m}^{-}\left(t\right)$. They called those corrupted primaries and we use their terminology. From what we have seen above, when a model has just 11 reflectors, the number of events in $h_{10}^{-}\left(t\right)$ is 1024 and only 11 of them are the desired primaries. All the other 1013 will be present in the same time window, overlap the physical primaries and their large number will outweigh their individual small strength. Hence, it is useful to not neglect the non‐physical primaries. Robinson and Treitel (1978) gave a backward recursion scheme that uses the reverse of equations (9) and (10), given by

(32) $$\begin{array}{c} \left(1-r_{n}^{2}\right)h_{n-1}^{+}\left(t\right)=h_{n}^{+}\left(t\right)-r_{n}h_{n}^{-}\left(t_{0n}-t\right), \end{array}$$

(33) $$\begin{array}{c} \left(1-r_{n}^{2}\right)h_{n-1}^{-}\left(t\right)=h_{n}^{-}\left(t\right)-r_{n}h_{n}^{+}\left(t_{0n}-t\right). \end{array}$$

With these equations, we need to start in the bottom and work our way up. Backward recursion implies that the data are used to compute the filter that corresponds to the bottom reflector and find the reflection coefficient $r_{m}$ directly from the latest event in the up‐going filter $h_{m}^{-}\left(t\right)$. We then use that inversion result to compute the filters that corresponds to next higher reflector and find the reflection coefficient in the same way as the previous one. Hence, the data are used only to compute the filters $h_{m}^{\pm }\left(t\right)$ and each reflection coefficient is found directly from the up‐going part of the filter after each recursion step. To find an equation from which $h_{m}^{+}\left(t\right)$ can be found from the reflection response, we convolve equation (5) with the fundamental wave field $h_{m}^{+}\left(t\right)$ This leads to

(34) $$\begin{array}{c} h_{m}^{+}\left(t\right)=\Phi \left(t\right)*h_{m}^{+}\left(t\right)+t_{0m}T\left(t_{0m}-t\right), \end{array}$$

where Φ(t) denotes the autocorrelation of the reflection response. Note that equation (34) is equal to equation (22) for n=m, but written such that $h_{m}^{-}\left(t\right)$ is avoided. This is possible only for n=m, because then $h_{m}^{-}\left(t\right)=R\left(t\right)\times h_{m}^{+}\left(t\right)$. The second term in the right‐hand side of equation (34) is zero for positive times. We write the down‐going fundamental wave field as $h_{m}^{+}\left(t\right)=\delta \left(t\right)+h_{m;m}^{+}\left(t\right)$, where $h_{m;m}^{+}\left(t\right)$ contains all unknown waves that are emitted after the initial impulse. Because this is a causal function, we evaluate equation (34) for positive times only. This leads to

(35) $$\left[h_{m;m}^{+}\left(t\right)-\Phi \left(t\right)*h_{m;m}^{+}\left(t\right)\right]H\left(t-\varepsilon \right)=\Phi \left(t\right)H\left(t-\varepsilon \right).$$

This equation can be solved for $h_{m;m}^{+}\left(t\right)$ for all positive times available. Once this wave field is known, equation (7) is used to determine $h_{m}^{-}\left(t\right)$. Within that wave field, the last event contains the local reflection coefficient of the bottom reflector in the data. It is found as

(36) $$\begin{array}{ccc} r_{m}\delta \left(t-2t_{0m}\right) & = & h_{m}^{-}\left(t\right)[H\left(t-2t_{0m}+\varepsilon \right)\\ & & -\;H(t-2t_{0m}-\varepsilon )], \end{array}$$

from which $r_{m}$ and the two‐way travel time, $2t_{0m}$, are known. With this information, the scheme then determines $h_{m-1}^{\pm }\left(t\right)$ from equations (32) and (33). The last event in $h_{m-1}^{-}\left(t\right)$ contains the reflection coefficient $r_{m-1}$ at its two‐way travel time $2t_{0(m-1)}$. In this way, we recursively move upward in the data until the local reflection coefficients and two‐way travel times of all reflectors are computed. Impedances are obtained using equation (31) and the inversion is complete.

### Marchenko impedance inversion by non‐recursive filtering

Equation (35) resembles equation (28) remarkably well, even though the equations have quite different interpretations and meaning. Equation (28) uses only the reflection response and can be solved for the wave field k(0,ζ,t) within its time window of validity for any constant value of ζ. The impedance at that particular vertical travel time is directly obtained by evaluating (Berryman and Greene 1980)

(37) $$Z\left(\zeta \right)=Z\left(0\right)\left[1+\int_{t=-\zeta }^{\zeta }k\left(0,\zeta ,t\right)dt\right].$$

The value 1 in the right‐hand side comes from the fact that the initial impulse is not part of the wave field k(t) but contributes to the impedance. With this evaluation the inversion is complete. The integral can be seen as a Fourier transformation at zero frequency, which is the only frequency used to compute the impedance.

### Marchenko‐type inversion by non‐recursive filtering

Equations (24) and (25) can be used in two ways to perform the inversion. For both, the first step is to use them together to simultaneously solve for $h^{\pm }\left(0,\zeta ,t\right)$ for any fixed value of ζ, let us say $t_{0n}<\zeta_{a}<t_{0(n+1)}$, by limiting the evaluation of the equations to $0<t<2\zeta_{a}$. These are given by

(38) $$\begin{array}{c} \int_{t^{\prime }=0}^{t}R_{0m}\left(t-t^{\prime }\right)h^{+}\left(0,\zeta_{a},t^{\prime }\right)dt^{\prime }=h^{-}\left(0,\zeta_{a},t\right), \end{array}$$

(39) $$\begin{array}{c} \int_{t^{\prime }=t}^{2\zeta_{a}}R_{0m}\left(t^{\prime }-t\right)h^{-}\left(0,\zeta_{a},t^{\prime }\right)dt^{\prime }=h^{+}\left(0,\zeta_{a},t\right), \end{array}$$

Equations (38) and (39) are the same as equations (24) and (25) in Slob *et al*. (2014) with a time shift for the fundamental wave fields. The first method retrieves the reflection coefficient $r_{n}$ as the last event in $h^{-}\left(0,\zeta_{a},t\right)$ at its physical two‐way travel time, $2t_{0n}$, or,

(40) $$r_{n}\delta \left(t-2t_{0n}\right)=h^{-}\left(0,\zeta_{a},2t_{0n}\right).$$

We observe that this is similar to the backward recursive scheme, but here it is not recursive and we can evaluate at any vertical travel time. Of course, from one reflection coefficient, only the impedance ratio can be obtained, cf. equation (31). The second method uses $h^{\pm }\left(0,\zeta_{a},t\right)$ to evaluate equation (24) for larger values of *t*. The first event in $t^{+}\left(\zeta_{a}\right)G^{+}\left(0,\zeta_{a},t-\zeta_{a}\right)$ will be the next primary reflection event in the data, it will have its physical amplitude, $t_{0n}^{+}t_{0n}^{-}r_{n+1}$, and will be present at its two‐way travel time, $2t_{0(n+1)}$. Equation (15) is evaluated to determine the factor $t_{0n}^{+}t_{0n}^{-}$ after which the reflection coefficient $r_{n+1}$ is known. It is given by

(41) $$r_{n+1}\delta \left(t-2t_{0(n+1)}\right)=\frac{t^{+}\left(\zeta_{a}\right)G^{+}\left(0,\zeta_{a},t_{0(n+1)}\right)}{t_{0n}^{+}t_{0n}^{-}}.$$

We observe that this step is similar to the forward recursive scheme, but here it is not recursive and we can perform this step at any vertical travel time.

In this method, we combine the ideas of the forward and backward recursive schemes and make it non‐recursive by using the idea behind the Marchenko equation. As a consequence, only this method can be performed with finite frequency bandwidth data in a target‐oriented manner. Once we have made the first non‐recursive step at a chosen vertical travel time, we can explore the target zone by using the recursive relations of equations (32) and (33) to move in upward direction, or by using the recursive relations of equations (9) and (10) to move in downward direction. The benefit of this possibility is illustrated in the next section for 3D wave fields in a horizontally layered medium.

## TARGET‐ORIENTED INVERSION WITH 3D FINITE FREQUENCY BANDWIDTH WAVE FIELDS

When one shot gather of the reflection response of a horizontally layered medium is available, we transform it to the horizontal‐slowness intercept‐time domain, with radial slowness, *s*, and intercept time, τ. We assume that the up‐going pressure is known at the acquisition surface together with the source time signature, W(t), which we assume to be zero phase for simplicity. The source time signature has a finite‐frequency bandwidth and a zero mean. The Ricker wavelet used in the above examples qualifies. Real source time signatures often have smaller bandwidths than the Ricker wavelet. The up‐going pressure is then given by

(42) $$p^{-}\left(s,\tau \right)=\int_{t^{\prime }=0}^{\tau }R\left(s,\tau -t^{\prime }\right)W\left(t^{\prime }\right)dt^{\prime }.$$

The functions $t^{+}\left(s,\zeta \right)G^{\pm }\left(0,\zeta ,s,\tau \mp \zeta \right)$ convolved with the wavelet are denoted $P^{\pm }\left(0,\zeta ,s,\tau \right)$. Equations (38) and (39) are now written as

(43) $$\begin{array}{ccc} & & \int_{t^{\prime }=0}^{\tau }p^{-}\left(s,\tau -t^{\prime }\right)h^{+}\left(0,\zeta ,s,t^{\prime }\right)dt^{\prime }\\ & & \;=\int_{t^{\prime }=0}^{\tau }h^{-}\left(0,\zeta ,s,\tau -t^{\prime }\right)W\left(t^{\prime }\right)dt^{\prime }, \end{array}$$

(44) $$\begin{array}{ccc} & & \int_{t^{\prime }=\tau }^{2\zeta }p^{-}\left(s,t^{\prime }-\tau \right)h^{-}\left(0,\zeta ,s,t^{\prime }\right)dt^{\prime }\\ & & \;=\int_{t^{\prime }=0}^{\tau }h^{+}\left(0,\zeta ,s,\tau -t^{\prime }\right)W\left(t^{\prime }\right)dt^{\prime }, \end{array}$$

and the equations can be solved for each value of *s* separately. Note that the filters remain impulse response functions, within the bandwidth of the data. These two equations can be solved for $h^{\pm }\left(0,\zeta ,s,\tau \right)$ at any chosen vertical travel time, ζ, at any value of *s*. Suppose we solve the equations for several values of *s* and for $\zeta =\zeta_{a}$ and $t_{0(n-1)}<\zeta_{a}<t_{0n}$. Then we know $h^{\pm }\left(0,\zeta_{a},s,\tau \right)$ and the latest event in $h^{-}\left(0,\zeta_{a},s,\tau \right)$ is the event with amplitude $r_{n-1}\left(s\right)$ and intercept time $\tau_{0(n-1)}\left(s\right)=t_{0}q_{0}\left(s\right)+t_{1}q_{1}\left(s\right)+\cdots +t_{n-1}q_{n-1}\left(s\right)$, in which $q_{n-1}=\sqrt{1-{\left(sc_{n-1}\right)}^{2}}$ is the normalized vertical slowness in layer n−1. Hence, the first inversion method obtains

(45) $$r_{n-1}\left(s\right)\delta \left(\tau -2\tau_{0(n-1)}\left(s\right)\right)=h^{-}\left(0,\zeta_{a},s,2\tau_{p(n-1)}\left(s\right)\right).$$

We can now move in upward direction using the backward recursion of equations (32) and (33), which remain valid by replacing $t_{0n}$ by $\tau_{0n}\left(s\right)$ for each value of *s* for propagating waves.

For the second method, we use the band‐limited version of equation (24) for later times and obtain $P^{+}\left(0,\zeta_{a},s,\tau \right)$. The earliest event in $P^{+}\left(0,\zeta_{a},s,\tau \right)$ is the reflection from $z_{n}$ and we know its intercept time $\tau_{n}\left(s\right)=\tau_{n-1}\left(s\right)+t_{n}q_{n}\left(s\right)$, but not yet the local reflection coefficient, because all transmission effects are still present. We use the band‐limited version of equation (25) to compute $P^{-}\left(0,\zeta_{a},s,\tau =0\right)$ and obtain the local reflection coefficient of the deeper reflector as (Slob *et al*. 2014; Wapenaar *et al*. 2014)

(46) $$r_{n}\left(s\right)=\frac{P^{+}\left(0,\zeta_{a},s,2\tau_{n}\left(s\right)\right)}{P^{-}\left(0,\zeta_{a},s,0\right)}.$$

We can move in downward direction using the forward recursion of equations (9) and (10), which remain valid by replacing $t_{0n}$ by $\tau_{0n}\left(s\right)$ for each value of *s* for propagating waves .

We now outline a different target‐oriented inversion procedure than given in Slob, Wapenaar and Treitel (2018). After one Marchenko solution and one Green's function computation, we know the intercept times inside layer *n* as $\tau_{n}\left(s\right)=\tau_{0n}\left(s\right)-\tau_{0(n-1)}\left(s\right)$, and the reflection coefficients $r_{n-1}\left(s\right)$ and $r_{n}\left(s\right)$. We first use the intercept time to recover the thickness $d_{n}$ of the layer. Suppose the ray parameter is sampled with K+1 samples as $s_{k}$ with k=0,1,2,…,K and $s_{0}=0$. The layer thickness is found from the intercept times and the slowness values. The general expression for the intercept time is given by

(47) $$\tau_{n}\left(s_{k}\right)=\frac{d_{n}}{c_{n}}\sqrt{1-s_{k}^{2}c_{n}^{2}},$$

with known $\tau_{n}\left(0\right)=d_{n}/c_{n}$ and we rewrite equation (47) as

(48) $$\tau_{n}^{2}\left(s_{k}\right)=\tau_{n}^{2}\left(0\right)-s_{k}^{2}d_{n}^{2},$$

which leads to

(49) $$d_{n}=\sqrt{\tau_{n}^{2}\left(0\right)-\tau_{n}^{2}\left(s_{k}\right)}/s_{k},$$

with k>0. We find the velocity in the layer from

(50) $$c_{n}=d_{n}/\tau_{n}\left(0\right).$$

With this velocity we invert the reflection coefficient $r_{n-1}$ for $c_{n-1}$ and $\rho_{n}/\rho_{n-1}$ is obtained as final parameter. The reflection coefficient is written as

(51) $$r_{n-1}\left(s_{k}\right)=\frac{b_{n-1}-\sqrt{a_{n-1}\left(s_{k}\right)}}{b_{n-1}+\sqrt{a_{n-1}\left(s_{k}\right)}},$$

where $b_{n-1}$ is ratio of the impedances at the two sides of the reflector and is obtained from $r_{n-1}\left(0\right)$ as

(52) $$b_{n-1}=\frac{\rho_{n}c_{n}}{\rho_{n-1}c_{n-1}}=\frac{1+r_{n-1}\left(0\right)}{1-r_{n-1}\left(0\right)},$$

and $a_{n-1}$ is the ratio of the squared *q*‐factors given by

(53) $$a_{n-1}\left(s_{k}\right)=\frac{q_{n}^{2}\left(s_{k}\right)}{q_{n-1}^{2}\left(s_{k}\right)}=b_{n-1}^{2}{\left(\frac{1-r_{n-1}\left(s_{k}\right)}{1+r_{n-1}\left(s_{k}\right)}\right)}^{2}.$$

Because $c_{n}$ is known, we find $c_{n-1}$ from

(54) $$c_{n-1}=\frac{\sqrt{a_{n-1}\left(s_{k}\right)-q_{n}^{2}\left(s_{k}\right)}}{s_{k}\sqrt{a_{n-1}\left(s_{k}\right)}}.$$

Now both velocities are known and the density ratio $\rho_{n}/\rho_{n-1}$ can be computed from equation (52). Because $r_{n}$ is also known, we can repeat this analysis and find $c_{n+1}$ and $\rho_{n+1}/\rho_{n}$. With this result the inversion is complete. This can be repeated by performing a forward or a backward recursion step, in which case we would continue with the method of Kunetz or Robinson and Treitel, respectively, without having to perform more Marchenko steps.

## NUMERICAL EXAMPLE

The methods described above are exact methods for piecewise constant one‐dimensional layered models. With impulse reflection response data they return all layer impedance values without error. When we use finite frequency bandwidth reflection response data, by convolving the impulse reflection response with a Ricker wavelet, Marchenko impedance inversion does not work anymore. When noise is added to the data the backward recursion method soon does not work anymore. The primary reason is that the autocorrelation of the reflection response is used to compute the fundamental wave field. The overall noise amplitude in the down‐going part of the fundamental wave field that we obtain is too high to estimate the up‐going part of the fundamental wave field to start the inversion in the bottom of the model. We do not show results for these two methods. We illustrate how the method of Kunetz (KI), of equation (30), and the Marchenko‐type method (MI), of equation (40), perform on computed data with multiplicative noise in a model with 12 reflectors. Once the reflection coefficients and their two‐way travel times are obtained, we use equation (31) recursively to compute the impedance as a function of two‐way travel time. Using the same model, we show how well the target‐oriented Marchenko‐type inversion (TOMI) scheme works.

The model information is given in Table 2. The source emits a 30 Hz Ricker wavelet with which we compute the reflection response. For illustrating TOMI, we compute the response for 10 different slowness values and use multiplicative noise. We compute the noise in the frequency domain with a base amplitude of 0.1 and a random phase after which it is multiplied by and added to the reflection response. This is equivalent to creating a random white noise trace in time domain, convolving it with the reflection response and then adding it to the reflection response. The time domain noise has extreme values of ±0.009. We compute 1000 different noise realizations and add each to the reflection response. We have tested the inversion schemes with only additive noise, and with both additive and multiplicative noise, and the results statistics are the same as the results shown here for multiplicative noise.

**Table 2:** Values for the medium parameters in the model for the numerical example

Layer number	Velocity (m/s)	Density (kg/m^3^)	Thickness (m)	$Z/Z_{0}$ (−)
0	1700	1430	∞	1.000
1	2300	2750	64.0	2.602
2	1900	2000	49.2	1.563
3	1700	1430	153.6	1.000
4	2100	1750	216.4	1.512
5	3200	2930	335.9	3.857
6	2000	1750	55.7	1.440
7	2100	2110	154.8	1.823
8	3300	1970	85.4	2.674
9	2500	2110	199.0	2.170
10	3000	2110	162.6	2.604
11	2500	2250	147.7	2.314
12	2900	2300	∞	2.744

For each of these 1000 datasets, we compute inversion results with KI and MI. In the inversion, we first compute the reflection coefficients for each method. For both methods, we perform blind inversion as a fully automated process. The only model assumption is that all layers have constant parameters. We do not assume a predetermined number of layers, but we assume that the layers are thick enough to prevent resolution problems to occur. The 30 Hz Ricker wavelet is 62 ms long to an amplitude level of 0.0023. This means we assume that the two‐way travel time inside each layer is 62 ms or more. In the model we use in this example, four layers have a two‐way travel time between 51 ms and 56 ms (layer numbers 1, 2, 6 and 8). In the 51 ms window, the smallest wavelet amplitude is 0.04, which is more than four times the noise level.

The KI method is implemented as follows. We search the extreme value in the reflection response within the first 62 ms time window. When this value is at or below the noise level, we assume it is noise and search again by shifting the search window by one window length. When this value is above the noise level, we assume it is part of a reflection and search for another extreme value within the same window length but with half a window length shift. When that extremum is larger than the one found earlier, its location and value provide the time location and reflection coefficient value. Otherwise the location and value of the previously found extremum provide the time location and reflection coefficient value. We then apply the forward recursion step and search the resulting trace by shifting the time search window by one window length from the detected reflector location. This is repeated until the end time is reached. With this method, the number of detected reflectors has a mean value of 12.1, a maximum of 19 and a standard deviation of 0.4.

The MI method is implemented in two steps. We first solve the coupled Marchenko‐type equations to obtain the primary reflectivity as a function of travel time. The resulting trace contains only primary reflections convolved with the Ricker wavelet. On that trace we proceed as we did in KI but now without the need to use the recursive steps, because all multiples have been eliminated already. We use a threshold of 0.04 to decide whether an extremum is part of a reflection or noise. This higher level can be used because in the trace to be analysed the transmission effects have been eliminated and true reflectivity amplitudes are present. The number of detected reflectors has a mean of 12.5, a maximum of 21 and a standard deviation of 1.1. For both results, we compute the constant impedance values for each layer for all travel times within that layer to plot the results.

**Figure 8:**
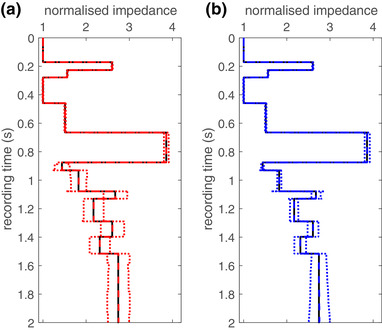
The retrieved mean impedance value and at one standard deviation for KI (a) and MI (b). The mean values are normalized to the impedance of the upper half space. The true normalized impedance is shown by solid black lines, the mean impedances by dashed lines and at one standard deviation by dotted lines, in red for KI and in blue for MI.

Figure 8 shows the mean normalized impedance values and the value at one standard deviation away from the mean as a function of travel time as obtained from KI in Fig. 8(a) and from MI in Fig. 8(b). The impedance of the upper half space is used as normalization factor. The true values are shown in black solid lines. The KI retrieved values are shown in red with the mean by a dashed line and the values at one standard deviation by dotted lines. The MI results are shown in a similar way in blue colours. It can be seen that the mean values are quite accurately obtained for both methods. The mean value errors are below 0.5% for KI and MI. The mean timing errors are below ±4 samples for both methods. In Fig. 8(a), we can see that the standard deviation of the impedance values suddenly jumps up in layer with number 6, which is where the first error in the arrival time estimations occur. The standard deviation grows in the next two layers but then stabilizes until the end of the trace. In Fig. 8(b), we can see that the standard deviation of the impedance values slowly grows and continues to grow after the last reflector is found. From reflector 6 onward in this model, KI shows larger standard deviations than MI but at the end of the trace the standard deviations are the same.

**Figure 9:**
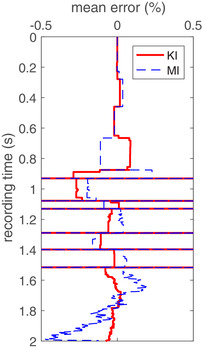
The error in the mean values of the normalized impedance as a function of travel time for KI (red solid) and MI (blue dashed). The horizontal lines in both results indicate large errors (<10%) arising from small errors in the retrieved travel times of the reflectors.

Both methods retrieve the impedance of each layer with acceptable errors in the mean values of the retrieved results. This is illustrated in Fig. 9 where it can be seen that the erratic behaviour of the error in the impedance between 1.5 seconds and 2 seconds is the effect of the noise that causes the methods to detect a reflector. It looks so erratic because at every noise realization those non‐physical reflectors are detected at slightly different times. They do not create large changes in the impedance. Note that the error axis is bounded by ±0.5% error. The horizontal lines are spiky errors that occur after 0.8 seconds. They all coincide with small timing errors of the reflector locations. These errors are similar in both methods, below 10% in amplitude and less than 4 ms in time location.

**Figure 10:**
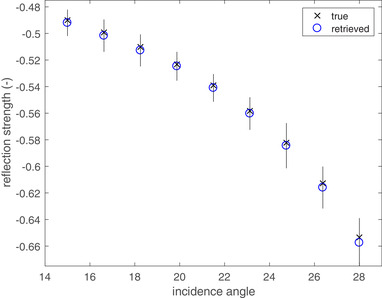
The true and mean retrieved reflection amplitude *r*_5_ as a function of incidence angle; the vertical lines indicate the standard deviation of the retrieved results.

The coupled Marchenko equations can be used at any two‐way travel‐time value for TOMI. We assume that the reflection response is obtained such that we have data at zero incidence, $\varphi_{0}=0$, and at nine angles of incidence, $\varphi_{k}$, in the upper half space from 15° to 28°, such that the radial slowness is given by $s_{k}=\sin\left(\varphi_{k}\right)/c_{0}$. We choose the same noise levels and compute 1000 realization of the reflection responses at these angles of incidence. For each dataset, we perform the inversion as outlined in equations (49)–(54) by computing the mean value using all values for the radial slowness. We choose the truncation time values $\zeta_{\alpha }\left(s_{k}\right)$ inside layer number 6 (n=6). The latest event in $h^{-}\left(0,\zeta_{\alpha },s,\tau \right)$ has amplitude $r_{5}\left(s_{k}\right)$ and the time of arrival is $2\tau_{05}\left(s_{k}\right)$. As explained in the text above equation (46), we then use the band‐limited version of equation (24) for $\tau \left(s\right)>\zeta_{\alpha }\left(s_{k}\right)$ and find the time of arrival of the first event in $P^{+}$, which is equal to $2\tau_{06}\left(s_{k}\right)$. We now know the interval intercept time $\tau_{6}\left(s_{k}\right)=\tau_{06}\left(s_{k}\right)-\tau_{05}\left(s_{k}\right)=t_{6}q_{6}\left(s_{k}\right)$. The arrival times are not significantly influenced by the noise after one Marchenko step. This means that the layer thickness, *d*_6_ and the corresponding velocity *c*_6_ are found with mean value errors well below 1% and very small standard deviations. The true and mean retrieved reflection amplitude *r*_5_ from $h^{-}$ is shown as a function of incidence angle in Fig. 10. The vertical lines indicate the retrieved results within one standard deviation. The figure shows that the errors in the mean retrieved value and the standard deviation increase with increasing angle of incidence. The maximum error in the mean value is below 1% and occurs at 28°. The values at one standard deviation have an error below 3%. The numerical values are summarized in Table 3.

**Table 3:** Values for the medium parameters in the model and for the TOMI result

	*d*_6_ (m)	*c*_6_ (m/s)	*c*_5_ (m/s)	$\rho_{6}/\rho_{5}$ (‐)
True	55.7	2000	3200	0.594
Mean	55.9	2007	3209	0.595
Std	0.2	8	86	0.023

## DISCUSSION

The method of Kunetz (KI) is a forward recursive method and involves only convolutions of modelled fundamental wave fields with the data. The fundamental wave fields are modelled using equations (9) and (10). The only influence of the noisy data on the fundamental wave fields are the amplitude and arrival time of the estimated reflection coefficient. For this reason, this method is quite well behaved with regard to noise. Estimated reflection coefficients at shallower depth are used to compensate for the transmission effects for each new estimated reflection coefficient. Small amplitude errors lead to increasingly larger errors because of increasingly incorrect correction for transmission effects. Those errors and errors in the estimated arrival times of the reflections lead to incorrect times of the events in the fundamental wave fields. These lead in turn to incomplete cancelation of the multiple reflections. When the amplitudes of these remnant multiple refections are above the noise level, they lead to the estimation of non‐physical reflectors. The method suffers from increasing errors in estimating reflection coefficients and arrival times, because these are used to model fundamental wave fields, which in turn are used to detect and estimate the arrival time and reflection coefficient of a deeper reflector. These effects are minimal in the example shown here. The advantage of KI is that no equation has to be solved. It is consists of one convolution and modelling the fundamental wave fields at each step. This makes it a very fast method.

The Marchenko‐type inversion is a non‐recursive method that computes the fundamental wave fields by filtering the reflection response. Filtering involves a convolution and a correlation of the fundamental wave fields with the data. As a consequence, the noise in the data enters into the fundamental wave fields. The correlation of two noisy traces increases the noise in the result. This can be seen by the increase of the relative standard deviation in the impedance values obtained as a function of travel time with this method. An advantage of Marchenko‐type method (MI) is that it can be adapted to accommodate an unknown source wavelet, but then the down‐going part of the field must be known (Ravasi 2017). Another advantage of MI is that higher thresholds can be used to detect a reflector, because the local reflection coefficient values are present in the up‐going part of the fundamental wave field. This is necessary because the correlations make the noise levels grow with increasing travel time for the inversion. The larger standard deviation in the number of detected reflectors in MI than in KI is because the noise levels increase with increasing inversion times. Most of the reflectors detected in addition to the reflectors in the model occur after the last reflector of the model has been detected. Another implication of higher noise levels is that weaker reflectors whose reflections have large two‐way travel times will be missed by MI. This does not happen in the example shown here.

We evaluate the target oriented inversion method on noisy reflection responses for normal incidence and nine non‐zero values for the radial slowness. We use the interval intercept times at the available angles of incidence to find the value for the layer thickness for each noise realization. Then the velocity inside the layer is obtained from the layer thickness and the normal incidence intercept time. Estimating the intercept times of the reflections from the top and bottom boundaries of the layer can be done with high accuracy. This means that the estimate of the intercept times of the layer are very accurately obtained. For this reason, both the layer thickness and the velocity in the layer are obtained with high accuracy and small standard deviations. Figure 10 shows that the mean values of the retrieved reflection strength is very accurate for all values of the incidence angle and the largest standard deviation is below 3% of the reflection coefficient value. For this reason and the fact that *c*_6_ is accurately obtained from the intercept times, the mean value of the velocity in the layer number 5 and the density ratio of the layers 5 and 6 are very accurately obtained as well. Because of the increased standard deviation in the reflection coefficient, the standard deviation in the retrieved values of *c*_5_ is higher, but still acceptable at less than 3%. Given the lower sensitivity to noise of the Kunetz method, it seems a good idea to continue with this method recursively after one Marchenko step. The Marchenko method is already adapted to retrieve local reflectivity in three‐dimensional (3D) that could be used for inversion (Wapenaar *et al*. 2014). Another interesting option seems to investigate the method of Kunetz further to see whether it can be extended for use in a general 3D setting.

## CONCLUSIONS

We have discussed two recursive and two non‐recursive methods for computing the reflection coefficients of a discrete layered model. The recursive methods use one equation that involves the data and two model equations that are recursively used to compute the fundamental wave fields. The forward recursive scheme computes the reflection coefficient from the data. The backward recursive scheme computes the reflection coefficients from the up‐going part of the fundamental wave field. The non‐recursive methods use only equations that involve the data to compute the fundamental wave fields at any chosen travel time. The first non‐recursive method is the Marchenko impedance inversion method. The method first computes a particular sum of the up‐ and down‐going parts of the fundamental wave fields. It then computes the impedance directly from the zero‐frequency value of that sum of the fundamental wave fields. The second is a Marchenko‐type method that first computes the up‐ and down‐going parts of the fundamental wave field from the data and then computes the reflection coefficient. The first three methods were developed independently almost 60 years ago. We have shown that these were revived briefly several times during the six following decades, but remained seen as independent. We have shown from the underlying physics that all four methods are based on the concept of the fundamental wave field.

In the Marchenko‐type method, the two equations are solved as a coupled set of equations for a chosen vertical travel time. We can then proceed in two ways. The first is to find the local reflection coefficient directly in the up‐going part of the fundamental wave field. It is the coefficient of the reflector that has a vertical travel time less than but closest to the chosen time instant. The second is to compute the local reflection coefficient from the first event in the Green's function. The fundamental wave fields are used to correct its amplitude for two‐way transmission effects. Either way, this scheme leads to the possibility of performing target‐oriented inversion. We have shown for a one‐dimensional model that when data are available for several ray parameters, the non‐recursive target‐oriented inversion can provide layer velocity, layer thickness and the ratio of densities of two adjacent layers. The non‐recursive nature of this method makes it a good candidate for extension to laterally varying media. Its sensitivity to noise can create problems at large times. The method of Kunetz is not very sensitive to noise (additive and/or multiplicative) and is very fast compared with the Marchenko‐type method, although it can be implemented only in a recursive way. The theory of the Marchenko‐type method is available for three‐dimensional (3D) inversion. The concept of forward recursive filtering seems worth investigating to see whether it can be adapted for use in a general 3D setting.

## DATA SHARING

The data that are used to make the plots of Figs. 8, 9, 10 can be obtained from the first author on request.
